# Effects of ionizing radiation on organic volatile compounds from PEA protein isolate

**DOI:** 10.1016/j.heliyon.2023.e22658

**Published:** 2023-11-22

**Authors:** Estelle Fischer, Philippe Cayot, Rémy Cachon, Nathalie Cayot

**Affiliations:** University Bourgogne Franche-Comté, Institute Agro Dijon, PAM UMR A 02.102, F-21000 Dijon, France

**Keywords:** Ionizing radiation, Electron beam, Oxidation, Pea protein isolate, Volatile compounds, HS-SPME-GC-MS, Beany off-flavor

## Abstract

Food irradiation is a preservation technique and in respect with regulations, is applied to a limited number of products. Nevertheless, this technique could be interesting for products sensitive to heat treatment, and to limit alteration caused to their organoleptic characteristics. This study concerns the potential of ionization for vegetable proteins, to limit the damage on the sensory properties that can be caused by thermal treatments. The impact of β-ionizing was measured on the volatile compounds of five pea protein isolates. These isolates were subjected to ionizing radiation of 10 MeV electron beam and the volatile compounds were compared by SPME-GC-MS before and after the treatment. β-Ionization led to a major increase in the total amount of volatiles and to appearance of new compounds. We observed a strong increase in aldehydes, that were reported to be involved in pea off-flavor, and the appearance of dimethyl-disulfide, linked to sulfurous off-notes. Many of the compounds impacted by the treatment were linked to protein and lipid oxidations. Mechanisms explaining the impact of β-ionizing on lipids and protein oxidations were proposed.

## Highlights

• Electron-beam irradiation initiated protein and lipid oxidations on pea protein isolate.
• Some volatile compounds were newly formed due to oxidation.
• The chemical families of volatile compounds evolved differently.
• Mechanisms of protein and lipid oxidations caused by irradiation were presented.

## 1 Introduction

Vegetable proteins are in high-demand for their numerous positive effects [[1], [2], [3]]. Many studies are conducted on their use for vegetable protein-based products, such as meat substitutes [4]. Vegetable proteins are frequently used as powders to be conveniently incorporated to food formulations. The manufacturing of protein powders begins by the obtention of protein suspensions, like protein isolates or concentrates [[5], [6], [7]]. The process may comprise a pasteurization or sterilization step before drying. This helps to remove any pathogen or contamination flora and ensure the product safety for further uses [8]. However, thermal methods for food preservation can lead to detrimental effects, such as loss of nutritional properties, modification of sensory properties, and denaturation of proteins [9,10]. In fact, thermal treatments generate protein modifications, irreversible changes in protein structures up to denaturation and loss of functionality [11], which are detrimental for the use of protein isolates and concentrates.

Some non-thermal processes have been investigated and developed to avoid the negative impacts of heat-treatments on the product. These alternative processes can be high pressure processing, pulsed electric field, ultrasound, ozone treatment, or ionizing radiation [12,13]. Food irradiation techniques may use either non-ionizing radiations (UV, visible light, and infrared for example) or ionizing radiations (gamma rays, X-rays, electron beam). Food ionizing irradiation has been developed as a preservation technique, to destroy microbes and extend the shelf-life of a product, without adversely affecting the product [12]. It is noticeable that ionizing irradiations can be carried out on final products in transparent packaging. For the present case, protein powders may be subjected to irradiation, and, due to low molecular mobility (as compared with the one in suspensions of proteins) this can limit some degradation reactions.

Ionizing radiation implies the application of gamma, electron beam, or X-rays on food products [[13], [14], [15]]. The electron beam technique is named β−‾radiation, is very ionizing (easily creates protein radicals) but gives a low-penetration ray. Photons are going through the food material and interacts with the food molecules to form charged ions. These charged ions then quickly change into highly reactive free radicals, reacting with themselves and uncharged molecules [13,16]. The decontamination effect induced by ionizing radiation comprise direct and indirect radiation [13,16]. In direct action, ionizing causes DNA damage and thus the inhibition of microbial DNA synthesis. In indirect radiation, cell lysis happens through the production of reactive water molecules such as hydrogen (H°) and hydroxyl radicals (OH°): RH + ionization → R° + H° and H_2_O ionization → H° + HO°. R°, H° and HO° are radicals, chemical species that possess a single electron (and not electron doublet, bonding, or non-bonding doublet). Radicals are very reactive species, more powerful than nucleophile species (that have one or several non-bonding doublet).

The use of ionizing radiation has shown great potential to ensure food safety, extending the product shelf-life and reducing food losses, by the removal of pathogenic and spoilage micro-organisms [17,18]. This technique was reported as safe towards the quality of the food-product, as leading to minimal sensory or nutritional modifications [16] and safe toward the health of the consumers [14]. However, discussions are still open about the impact of ionizing radiations on food products, as other studies have shown negative sensory impacts such as rotten or bloody off-odors on meat [19]. Modifications of the product through irradiation, such as lipid oxidation, radiolytic degradation of amino acids and therefore the production of new volatile compounds, are linked to these sensory modifications [20,21]. In particular, volatile sulfur compounds have been designated as playing a crucial role in the off-odor of irradiated products, called the “irradiation odor” [22].

With the debate still open, the international agencies, such as WHO (World Health Organization), FDA (Food and Drug Administration) and IAEA (International Atomic Energy Agency), have established a maximum exposition dose of 10 kGy for food products [13,16]. The Codex Alimentarius approved three ionizing radiations for the treatment of food products, gamma rays emitted through cobalt-60 or cesium-137, accelerated electrons that does not exceed 10 MeV and X-rays at a maximum energy of 5 MeV [14,23,24].

The French regulation (Arrêté du 20 août 2002 relatif aux denrées et ingrédients alimentaires traités par ionization) details a limited list of food products that are authorized to be treated by ionizing radiation. In respect with this regulation, the treatment for leguminous protein isolates is not authorized for commercial uses.

With the authorized applications, the effect of ionizing radiation on food products was already studied on various products such as meat [19,20], fishery products [25], raw fruits and vegetable [12,18], or spices and nuts [14]. Nevertheless, there is a lack of information on the impact on other types of products in particular powdery product and vegetable proteins, at low humidity, with high protein content and low amount of lipids.

The present study aimed to investigate the impact of electron beam ionization on the organic volatile compounds of pea protein isolates, and to propose a highlight on the different oxidation mechanisms that may occur in the product. Electron beam ionization was selected for its reported strong impact on reducing the pathogenic flora and its low sensory impact. Analysis of the volatile compounds was chosen to determine the possible sensory impact on the product and to trace the impact of ionization on oxidation of the food matrix, with special attention accorded to protein-oxidation products such as sulfurous compounds or lipid-oxidation products such as aldehydes. First, five different pea protein isolates with different profiles in volatile compounds were selected and subjected to ionizing radiation consisting of 10 MeV electron beam. Then, the volatile compounds were compared by SPME-GC-MS before and after the ionizing treatment. Finally, mechanisms of oxidation reactions inside the product and their impact on the volatile compounds of pea protein isolates were proposed.

## 2 Material and methods

### 2.1 Pea protein isolates

Different samples of spray-dried pea protein isolates (PPIs) were supplied by Roquette Frères S.A. Each PPI had a protein content of 85 % m/m, composed mainly of globulins, a water content of 7 % and a lipid content of 9 % m/m with a fatty acid profile comprising palmitic, oleic, linoleic, linolenic ad stearic acids. Five PPIs were selected to have different profiles in volatile compounds. During the short time before and after the ionizing treatment, samples were stored in sterile polypropylene tubes at 7 °C to limit microbial growth.

### 2.2 Raw material treatment, ionizing radiation

Samples were subjected to ionizing radiation treatment using an electron accelerator, isolated by concrete walls, thus concentrating the electron beam (β-ray beam) on the product. Samples were packed in polyethylene bags and had a maximum thickness of 1 cm. They were placed directly on a conveyer belt and forwarded under the electron accelerator. Ionization is then conducted with beta rays, at 10 MeV in one beam.

### 2.3 Analysis of volatile compounds by HS-SPME-GC-MS

The volatile compounds extraction was done using headspace solid phase micro-extraction and the analysis using gas chromatography coupled with mass spectrophotometry (HS-SPME-GC-MS). Volatile compounds analysis was run in triplicates for each sample, before and after treatment, using a method optimized for PPI [26].

A 0.2 g PPI sample was weighted directly in a 20 mL extraction vial (VA201) capped with septum caps (18 mm caps, 8 mm PTFE/silicon septum, SACA001), all purchased from JASCO (France). Distilled water was added to obtain a 2 mL suspension at 10 % (w/v) and a liquid/gas ratio of 2/18 (v/v). A SPME device containing a 1 cm fused-silica fiber coated with a 50/30 μm thickness of DVB/CAR/PDMS (divinylbenzene/carboxen/polydimethylsiloxane) was used for HS-SPME extraction. This fiber was selected to ensure the best extraction of a diversity of volatile compounds. The fiber (24 Ga 50/30 μm, for manual holder, 3 pK, 57328-U) was purchased from Sigma and used with a manual fiber holder. The extractions were carried out in an electro thermal magnetic stirrer with a water bath (MS-H-Pro+, DLAB) to ensure a homogeneous temperature and constant agitation for the sample and headspace. The fiber was conditioned before analysis by heating it in the gas chromatograph injection port at 270 °C for 30 min, according to the manufacturer's specifications. Equilibrium step and extraction step were conducted both at 40 °C with agitation at 350 rpm in the dark. The equilibrium time was 30 min and the extraction time, exposure of the fiber in the headspace of the vial, was 60 min.

An HP 6890 Series Gas Chromatograph (Hewlett-Packard, Palo Alto, CA, USA) equipped with an HP 5973 Mass Selective Detector (Agilent Technologies, Palo Alto, CA, USA) (Quadrupole) was used with a DB-WAX column (30 m ╳0.32 mm ╳ 0.25 μm, 123–7032, Agilent, J&W Scientific, Santa Clara, United States) to analyze the compounds of interest. The SPME fiber was desorbed and maintained in the injection port at 250 °C for 5 min. The sample was injected in split mode, with a purge flow of 140 mL/min at 0 min to generate sharp, well-separated peaks on the chromatograph. Helium was used as a carrier gas at 1.4 mL/min with a linear velocity of 43 cm/s. The programmed temperature, selected from preliminary trials, was isothermal at 40 °C for 3 min, raised to 100 °C at a rate of 3 °C/min, and then raised to 230 °C at a rate of 5 °C/min and held for 10 min. The total run time was 59 min. The ionization source and transfer line temperatures were set respectively at 230 °C and 190 °C.

The mass spectra were obtained using a mass selective detector with an electron impact voltage of 70 eV in full scan mode over the range *m*/*z* 29 to 400. Compounds were identified by comparing their mass spectra with NIST 08 (National Institute of Standards and Technology), Wiley, and INRA libraries, with a low integration limit of 50,000 in peak area, allowing the best peak identification.

### 2.4 Semi-quantification method

Ten compounds of interest were semi-quantified in the PPI, due to their involvement in the ‘beany’ off-flavor [[27], [28], [29], [30]]. The following standards were purchased from Sigma-Aldrich: hexanal (98 % purity, CAS 66-25-1), nonanal (>98 %, CAS 124-19-6), *trans*-2-nonenal (97 %, CAS 18829-56-6), 3-methylbutanal (97 %, CAS 590-86-3), 1-octen-3-ol (98 %, CAS 3391-86-4), 3-octen-2-one (98 %, CAS 1669-44-9), 2-pentylfuran (98 %, CAS 3777-69-3), benzaldehyde (99 %, CAS 100-52-7), 2,5-dimethylpyrazine (98 %, CAS 123-32-0) and 1-hexanol (98 %, CAS 111-27-3). An external calibration method, previously optimized for PPI [26] was used. The calibration curves of each of the ten compounds were obtained for concentrations ranging from 0.001 to 2.5 ppm, in distilled water. The amount of each compound in the sample was calculated as in the following example with hexanal for PEA1 before ionization. Semi-quantification steps were as following, with a and b from the calibration curve of hexanal (a = slope, b = intercept of the regression):

(1) AreaHexanal=5303819A.U.

(2) $${\left[\text{Hexanal}\right]}_{\text{in}\;\text{the}\;\text{assay}}\;\left(\mu g/\text{mL}\right)=\left(\text{Area hexanal}-b\right)/a=\left(5303819-157037\right)/1\times \;10^{7}=0.51\;\mu g/\text{mL}$$

$${\left[\text{Hexanal}\right]}_{\text{in}\;\text{the}\;\text{sample}}\left(\mu g/g\right)={\left[\text{Hexanal}\right]}_{\text{in}\;\text{the}\;\text{assay}}\left(\mathrm{\mu g}/\text{mL}\right)\;\times {\text{V}}_{\text{solution}}\left(\text{mL}\right))/{\text{ m}}_{\text{sample}}\left(g\right)\;\;$$

(3) $$=\left(0.51\times 2\right)/0.2568=4.01\mu g/g\;$$

(4) $$m{\left[\text{Hexanal}\right]}_{\text{in}\;\text{the}\;\text{sample}}\left(n=3\right)=4.1\pm 0.2\mu g\;\text{of}\;\text{hexanal}/g\;\text{of}\;\text{PPI}$$

### 2.5 Statistics/data analysis

The statistical treatment was processed using the software Statgraphics® Centurion XVII, version 17.1.04. (StatPoint Technologies, Inc., Warrenton, Virginia, USA), followed by Tukey's test with a 5 % level of significance. Analysis of variance (ANOVA) was performed to determine significant differences between the samples for a given volatile compound and a give pea protein isolate.

## 3 Results and discussion

### 3.1 Profile in volatile compounds of pea protein isolate

Volatile compounds of PPI typically comprise aldehydes, alcohols, ketones, furans, alkanes, alkenes and some other minim compounds. A typical volatile compound composition of a PPI is presented in Table 1 **(PEA1**, **before ionization)**. In this example, 23 volatile compounds were found, with five aldehydes (pentanal, hexanal, nonanal, 2-heptenal, benzaldehyde), five alcohols (1-pentanol, 1-hexanol, 1-octanol, 1-octen-3-ol, 1-penten-3-ol), five ketones (2-heptanone, 2-nonanone, 3-octen-2-one, 3,5-octadien-2-one, 6-methyl-5-hepten-2-one), two furans (2-ethyl-furan, 2-pentyl-furan) and some other compounds (pentane, heptane, octane, styrene, toluene, methanethiol). All these compounds were already reported in the literature as being part of the volatile compounds profile of PPIs [26,27,31,32].

**Table 1:** Impact of ionization on volatile compounds of pea protein isolates, in Area/g of sample (n = 3). Legend: B = Before ionization, A = After ionization. In **bold**, volatile compounds produced probably by oxidation of amino acid residues of pea proteins. In italic with a star*, volatile compounds produced probably by oxidation of fatty acid residues of pea triacylglycerols (pea lipids). Increase of volatile compounds calculated on chromatographic areas measured before and after ionization and regrouped by chemical family, in % of area (n = 3).

		PEA 1		PEA2		PEA3		PEA4		PEA5	
Compounds	CAS	B	A	B	A	B	A	B	A	B	A
ALDEHYDES		+110 %		+110 %		+970 %		+140 %		+1200 %	
**Butanal, 2-methyl-**	000096-17-3	–	427,960	–	414,575	284,524	396,705	341,078	438,157	–	431,163
**Butanal, 3-methyl-**	000590-86-3	–	569,958	197,140	537,631	–	560,955	295,530	639,537	396,714	613,574
Pentanal	000110-62-3	871,885	1,495,838	1,264,114	1,859,405	–	1,170,193	776,770	2,226,003	652,283	1,871,126
*Hexanal	000066-25-1	21,163,905	28,616,701	23,518,000	30,417,369	2,852,496	26,699,783	19,644,993	34,321,557	2,199,258	18,494,649
Heptanal	000111-71-7	–	–	–	–	–	–	265,746	–	–	–
*Octanal	000124-13-0	–	1,089,073	–	1,757,824	–	1,501,109	2,102,247	3,197,747	–	1,691,998
*Nonanal	000124-19-6	1,989,471	3,004,689	3,113,424	3,763,894	868,215	3,816,838	8,043,062	7,424,313	303,303	5,302,296
*Decanal	000112-31-2	–	–	79,018	–	–	–	478,559	298,267	–	–
*2-Hexenal, (E)-	006728-26-3	–	276,001	273,992	269,402	152,003	372,996	393,323	583,447	209,224	546,619
2-Heptenal, (Z)-	057,266-86-1	65,587	852,887	258,955	945,609	200,801	978,006	614,449	1,772,018	191,008	1,587,506
*2-Octenal, (E)-	002548-87-0	–	–	194,245	–	70,212	–	425,051	–	83,694	–
2-Octenal, 2-butyl-	013,019-16-4	–	–	–	–	–	–	148,509	–	–	–
*2-Nonenal	018,829-56-6	–	–	–	–	–	–	347,761	82,787	–	–
**Benzaldehyde**	000100-52-7	1,434,309	12,711,310	1,691,364	12,739,539	1,290,641	14,211,412	2,470,657	23,097,865	989,228	22,439,817
Benzaldehyde, 4-propyl-	028,785-06-0	–	3,704,291	–	7,737,869	–	8,004,123	–	10,882,174	–	10,932,689
ALCOHOLS		+160 %		+210 %		+40 %		+130 %		+360 %	
1-Pentanol	000071-41-0	475,665	575,226	200,676	283,561	1,328,332	1,123,668	256,773	685,298	499,671	908,388
1-Hexanol	000111-27-3	732,452	814,844	181,178	335,698	23,226,550	8,050,579	996,250	1,558,002	5,661,884	17,536,478
1-Heptanol	000111-70-6	–	–	–	–	914,916	466,370	104,917	–	–	759,896
1-Octanol	000111-87-5	79,932	355,764	73,176	–	820,017	283,095	583,129	–	–	746,832
1-Nonanol	000143-08-8	–	–	–	–	1,025,448	391,733	–	–	–	–
*1-Octen-3-ol	003391-86-4	1,776,518	5,248,310	1,422,453	3,898,475	1,931,646	4,288,074	3,354,396	8,981,585	1,317,098	7,249,087
1-Penten-3-ol	000616-25-1	158,332	185,959	–	–	–	–	–	–	–	–
1-Hexanol, 2-ethyl-	000104-76-7	–	312,293	73,412	451,194	83,252	3,499,542	368,262	1,155,952	–	2,767,307
1-Butanol, 3-methyl-	000123-51-3	–	–	–	–	327,520	–	–	–	–	–
2-Hexen-1-ol, (E)-	000928-95-0	–	–	–	–	417,889	–	–	–	–	–
2-Octen-1-ol, (E)-	018,409-17-1	–	428,443	–	–	–	–	–	–	–	258,224
2-Decen-1-ol, (E)-	018,409-18-2	–	–	–	–	–	–	92,914	–	–	–
KETONES		+120 %		+20 %		+50 %		+40 %		+190 %	
Acetone	000067-64-1	–	605,307	76,300	567,558	–	516,465	94,510	588,216	–	–
2-Butanone	000078-93-3	–	633,094	–	809,951	–	792,834	77,969	878,969	–	883,469
2-Heptanone	000110-43-0	3,529,476	6,346,234	12,519,619	7,927,455	11,519,173	8,871,882	16,956,059	12,919,816	7,030,610	9,050,189
2-Heptanone, 4-methyl-	006137-06-0	–	–	–	304,444	–	193,821	–	309,415	–	237,333
*2-Octanone	000111-13-7	–	659,811	–	569,467	–	861,091	–	1,421,240	–	1,166,924
*3-Octanone	000106-68-3	–	389,174	–	248,632	–	408,297	308,171	724,878	–	627,681
2-Nonanone	000821-55-6	171,501	377,917	1,121,491	759,159	1,006,094	723,390	1,436,446	1,172,419	839,426	1,133,053
2-Decanone	000693-54-9	–	328,492	973,045	627,638	788,625	435,746	2,601,995	997,176	276,762	596,813
3-Octen-2-one	001669-44-9	68,756	641,542	301,626	1,565,908	666,413	3,259,198	789,115	4,264,775	278,162	4,458,456
3,5-Octadien-2-one, (*E*,*E*)-	030,086-02-3	3,199,727	5,269,552	1,865,777	5,352,533	3,512,119	8,778,356	5,937,789	15,814,952	2,392,602	13,109,453
5-Hepten-2-one, 6-methyl-	000110-93-0	497,794	647,579	333,488	458,557	563,179	691,791	733,354	807,226	309,492	713,226
1-hepten-3-one	000000-00-0	–	–	–	–	141,385	–	–	–	–	–
2,3-Octanedione	000585-25-1	–	–	1,145,800	1,455,195	–	210,819	526,193	1,941,510	737,155	1,643,105
FURANS		+10 %		+20 %		+10 %		+20 %		+120 %	
2-*n*-Butyl furan	004466-24-4	–	365,398	–	–	–	–	–	167,196	–	–
Furan, 2-ethyl-	003208-16-0	789,677	–	–	–	200,120	–	221,994	–	–	–
Furan, 2-pentyl-	003777-69-3	9,843,439	11,001,264	9,451,255	6,835,378	9,874,669	8,658,600	15,937,227	12,008,973	5,687,229	12,044,899
ALKANES		+280 %		+570 %		+400 %		+340 %		+510 %	
Pentane	000109-66-0	287,296	866,040	–	679,860	274,215	900,412	87,771	1,065,739	245,612	1,025,904
Heptane	000142-82-5	590,064	1,516,822	556,762	1,495,770	689,347	1,655,648	941,476	2,200,019	929,417	2,188,980
Octane	000111-65-9	1,637,975	4,174,858	1,585,955	3,552,958	1,957,730	3,380,393	2,337,472	4,507,307	1,866,583	4,172,438
Nonane	000111-84-2	–	878,936	–	876,703	–	823,577	–	801,648	–	859,664
Dodecane	000112-40-3	–	75,669	–	1,073,835	–	429,814	–	236,337	–	612,837
Heptane, 4-methyl-	000589-53-7	–	77,432	–	507,896	–	517,946	–	–	–	804,253
Octane, 4-methyl-	002216-34-4	–	456,666	–	1,650,575	–	1,697,265	–	946,715	–	2,364,023
Nonane, 2,6-dimethyl-	017,302-28-2	–	1,262,592	–	3,819,486	–	3,758,834	–	3,412,605	–	2,284,951
Decane, 3,6-dimethyl-	017,312-53-7	–	357,860	–	740,954	–	1,189,102	–	1,169,168	–	1,722,508
Decane, 3,7-dimethyl-	017,312-54-8	–	519,273	–	1,694,644	–	2,376,515	–	2,364,912	–	2,711,799
ALKENES		+4300 %		+6400 %		+2000 %		+2400 %		+4200 %	
Styrene	000100-42-5	169,674	–	455,253	100,716	1,574,343	334,330	981,087	1,501,451	1,050,100	556,898
**Toluene**	000108-88-3	79,089	10,403,718	–	6,560,380	307,718	5,223,433	507,120	7,689,104	–	6,892,888
**Benzene, 1,3-bis(1,1-dimethylethyl)-**	001014-60-4	–	1,404,978	–	15,242,894	–	27,485,379	–	22,815,847	–	29,556,377
**Benzene, propyl-**	000103-65-1	–	–	–	320,783	–	177,363	–	354,315	–	365,104
1-Octene	000111-66-0	–	467,541	–	349,837	–	343,545	–	356,497	–	447,140
2-Octene, (Z)-	007642-04-8	–	521,373	–	176,209	–	86,712	–	315,383	–	1,332,598
1-Heptene	000592-76-7	–	819,474	–	412,090	–	405,778	–	463,353	–	479,187
1-Undecene	000821-95-4	–	–	–	440,703	–	389,819	–	432,391	–	620,130
2,4-Dimethyl-1-heptene	019,549-87-2	–	1,096,454	–	3,843,295	–	3,763,288	–	1,767,280	–	4,151,210
OTHERS											
Pyrazine, 2,5-dimethyl-	000123-32-0	–	–	–	–	–	–	276,873	–	–	–
Methanethiol	000074-93-1	254,665	357,599	252,761	80,277	–	–	87,670	–	–	–
**Disulfide, dimethyl**	000624-92-0	–	288,735	–	744,205	–	874,320	–	760,523	–	1,108,603
2-Propenoic acid, 2-ethylhexyl ester	000103-11-7	–	–	–	1,002,276	–	6,001,565	–	3,005,755	–	8,138,459
1H-Inden-1-ol, 2,3-dihydro-2-methyl-	017,496-18-3	–	–	–	366,397	–	127,495	–	455,669	–	546,209

**Fig. 1:**
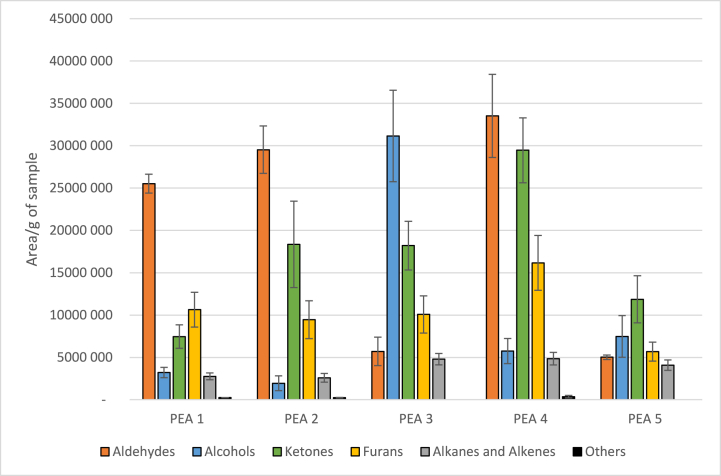
Profiles in volatile compounds of the initial different pea protein isolates. The amount of volatile compounds was measured using HS-SPME-GC-MS and data were regrouped by chemical families. Error bars represent standard deviations.

In the present study, we studied five different samples of PPI. Their profiles in volatile compounds (chemical families) were represented in Fig. 1. PEA1 and PEA2 were selected for their classic profile in volatile compounds (high amount of aldehydes, presence of alcohols, ketones and furans). PEA3 was selected for its high amount of alcohols and PEA4 for its high amount of aldehydes and ketones. Finally, PEA5 was selected for its low amount of total volatile compounds. These sample were selected to study the impact of ionizing β-radiation on different profiles in volatile compounds, to compare the impact of electron beam on already present compounds, and to determine if the initial profile has an impact on the evolution of volatile compounds in quantity and in quality.

### 3.2 Effect of ionizing radiation on the profiles in volatile compounds of pea protein isolates

**Fig. 2:**
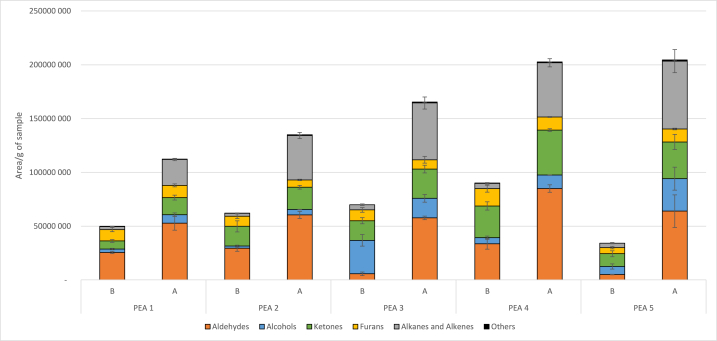
Impact of ionization on the different chemical families of volatile compounds found in pea protein isolates measured by HS-SPME-GC-MS. Legend: B = Before ionization, A = After ionization. Error bars represent standard deviations.

The impact of electron beam ionization on the volatile compounds of PPIs is presented in Table 1 with the area of each detected compound, in Table 2 with the amount of the ten semi-quantified compounds, and finally represented in Fig. 2 by chemical families. The amounts of the ten semi-quantified compounds were compared before and after ionization for each sample, and significant differences were reported in Table 2.

**Table 2:** Amounts of the semi-quantified volatile compounds before and after ionization, for the different samples of pea protein isolates, in μg of compound per g of sample (n = 3). Nd = not detected, Nq = not quantified, below the quantification limit. For each pea protein isolate and each volatile compound, different letters (^x,y^) indicate significant differences between the values according to ANOVA (p < 0.05).

Compound	CAS	PEA1		PEA2		PEA3		PEA4		PEA5	
		B	A	B	A	B	A	B	A	B	A
Hexanal	000066-25-1	4.1 ± 0.2^x^	5.6 ± 0.4^y^	4.6 ± 0.9^x^	5.9 ± 0.5^x^	0.4 ± 0.1^x^	5.2 ± 0.4^y^	3.8 ± 0.3^x^	6.71 ± 0.07^y^	0.3 ± 0.1^x^	3.6 ± 0.7^y^
Nonanal	000124-19-6	0.32 ± 0.06^x^	0.5 ± 0.2^x^	0.5 ± 0.2^x^	0.67 ± 0.03^x^	0.10 ± 0.05^x^	0.7 ± 0.3^y^	1.5 ± 0.7^x^	1.40 ± 0.08^x^	Nq^x^	1.0 ± 0.4^y^
2-nonenal	018,829-56-6	Nd	Nd	Nd	Nd	Nd	Nd	0.45 ± 0.02^x^	Nd^y^	Nd	Nd
3-methyl-butanal	000590-86-3	Nd	Nq	Nq	Nq	Nd	Nq	Nq	Nq	Nq	Nq
Benzaldehyde	000100-52-7	0.39 ± 0.06^x^	3.6 ± 0.8^y^	0.5 ± 0.2^x^	3.6 ± 0.4^y^	0.4 ± 0.2^x^	4.0 ± 0.9^y^	0.7 ± 0.2^x^	6.6 ± 0.3^y^	0.26 ± 0.06^x^	6 ± 1^y^
1-hexanol	000111-27-3	Nq	Nq	Nq	Nq	7 ± 1^x^	2 ± 1 y	Nqx	0.14 ± 0.03^y^	1.5 ± 0.9^x^	6 ± 1^y^
1-octen-3-ol	003391-86-4	0.15 ± 0.02^x^	0.50 ± 0.04^y^	0.11 ± 0.04^x^	0.36 ± 0.02^y^	0.17 ± 0.04^x^	0.40 ± 0.03^y^	0.31 ± 0.08^x^	0.87 ± 0.01^y^	0.10 ± 0.02^x^	0.7 ± 0.3^y^
3-octen-2-one	001669-44-9	Nd^x^	0.08 ± 0.02^y^	0.039 ± 0.002^x^	0.26 ± 0.02^y^	0.08 ± 0.03^x^	0.6 ± 0.2^y^	0.10 ± 0.04^x^	0.80 ± 0.03^y^	0.03 ± 0.02^x^	0.84 ± 0.04^y^
2-pentyl-furan	003777-69-3	6 ± 1^x^	6.3 ± 0.8^x^	6 ± 1^x^	4.2 ± 0.3^x^	6 ± 1^x^	5 ± 1^x^	9 ± 2^x^	6.8 ± 0.3^x^	3.6 ± 0.6^x^	6.9 ± 0.4^y^
2,5-dimethylpyrazine	000123-32-0	Nd	Nd	Nd	Nd	Nd	Nd	0.42 ± 0.08^x^	Nd^y^	Nd	Nd

By first looking at Fig. 2 it can be seen that ionization resulted globally in increased amounts of volatile compounds for each sample, that might lead to organoleptic modifications [33]. There was a strong increase in aldehydes, alkanes, and alkenes compounds. These effects were already reported as a result of the ionization process [34,35].

As observed in Table 1, the amount of almost each aldehyde that was initially present in the samples increased during the ionization process. Additionally, some new aldehydes appeared, like octanal, 2-methyl- or 3-methyl-butanal in PEA1, PEA2, PEA3 and PEA5. Aldehydes are generally derived from lipids oxidation [36,37] but can also arise from protein oxidation, as 2-methyl- and 3-methyl-butanal, respectively originating from isoleucine or leucine and valine [34,38].

In Table 2, a marked increase in the amount of each semi-quantified aldehyde can be observed, for example for hexanal, rising from 4.1 to 5.6 μg/g of sample in PEA1 or from 0.3 to 3.6 μg/g of sample in PEA5. Initially “not detected”, 3-methyl-butanal was present but “not quantified” after ionization. Benzaldehyde, coming both from lipid oxidation and from protein oxidation, can be seen as a marker of the protein oxidation. It increased from 0.3 to 0.7 to 3.6–6.6 μg/g of sample.

The same observations can be made with alkanes and alkenes, with both increasing amounts and increasing diversity after ionization (Table 1). At least six new alkanes and five new alkenes appeared in the samples after ionization. These compounds were coming from the protein oxidation due to the ionizing process, and were already reported in other studies [19,21,34,35]. Alcohols and ketones also increased in all the samples (except for alcohols in PEA3), and were reported as coming from lipids and proteins oxidations induced by the ionizing process [19,21,[33], [34], [35]]. For example, in Table 2, 1-octen-3-ol increased from 0.15 to 0.50 μg/g of sample in PEA1 and from 0.31 to 0.87 μg/g of sample in PEA4. Also, 3-octen-2-one increased from not detected to 0.08 μg/g of sample in PEA1 and from 0.10 to 0.80 μg/g of sample in PEA4.

Finally, dimethyl-disulfide appeared in all samples after ionization (Table 1). This volatile compound is characteristic of ionized products. It is typically found after an ionizing radiation step, and is an impacting compound in the “ionization odor” [21,33,35]. It is well-know that *S*-containing volatiles appear after ionization, coming from *S*-containing compounds like amino acids (methionine in the case of dimethyl-disulfide), through protein oxidation [19,34].

The different chemical families were not impacted similarly by ionization. The percentage of increase of the volatile compounds regrouped by chemical families were reported in Table 1. For example, alkanes and alkenes were the most impacted, with an increase of 280–570 % for alkanes and 2000–6400 % for alkenes. Then came the aldehydes. For samples with a high initial level of aldehydes (PEA1, PEA2, PEA4), an increase of approximately 100 % was observed and, for sample with a low initial level of aldehydes (PEA3, PEA5), an increase of approximately 1000 % was observed. Alcohols were impacted differently between samples, from 40 % in PEA3 to 360 % in PEA5. Ketones were the less impacted, with most samples below 100 % and furans almost not impacted except in PEA5. Dimethyl-disulfide only appeared in ionized samples. These results concerning the most impacted chemical families (alkenes, alkanes, and aldehydes) are in accordance with the scientific literature [21,34,35].

### 3.3 Discussion: effect of β-ionization

It was reported by Feng & Ahn (2016) that the volatile compounds, newly formed or whose concentration was strongly increased after β-ionization, were coming from lipid and protein oxidations. In a complex matrix with protein and lipids (chicken in the cited publication), hexanal and pentanal were reported to come from lipid oxidation [20]. In a mixture of lipid in emulsion, amino acids or protein, hexanal was reported as a volatile that reflected essentially lipid oxidation [39].

Feng & Ahn (2016) reported that the strong increase of alkenes, the increase of alkanes, aldehydes and ketones were mainly due to protein oxidation and the increase of content of volatile alcohols rose from lipid oxidation, even if lipid oxidation also produced aldehydes and ketones. So, hexanal and pentanal increase after irradiation might be due to protein and to lipid oxidations.

Feng et al. (2016) reported a study using irradiated pure amino acid esters that mimicked aminoacyl residues in protein. We can extrapolate their data to examine the results of Table 1 and conclude on protein oxidation.

Concerning aldehydes, 2-methyl-butanal (PEA 1, 2, 5) and 3-methylbutanal (PEA 1, 3) appeared or strongly increased after irradiation: 2-methyl-butanal was a product of oxidation of Isoleucine residues of proteins, and 3-methyl-butanal (also named isovaleraldehyde) was a product of oxidation of Leucine residues of proteins.

About ketones, the production of 2-butanone during irradiation for all PPI (except PEA4 where the concentration strongly increased) could also be due to the oxidation of Isoleucine residues of pea protein. The apparition or strong increase of toluene concentration after irradiation would find its origin in the oxidation of Phenylalanine and Tyrosine residues. The strong increase of content of benzaldehyde was due to the oxidation of Phenylalanine residue [21,40]. Volatiles such as benzene, 1,3-dimethyl benzene, 1,4-dimethyl benzene, isopropyl benzene, ethyl benzene were produced by irradiation due to protein oxidation [40].

In Table 1, the apparition of 1,3-bis(1,1-dimethylethyl)- benzene and propyl-benzene was probably due to protein oxidation.

The production of dimethyl sulfide could be due to the oxidation of Methionine residues according to the literature [21,39]. The appearance of this volatile sulfur compound was due to the strong sensitivity of *S*-atom to ionizing irradiation. In fact, *S*-atom is named “soft atom”, because it is a strongly polarizable big atom with a high number of electrons on the last layer (1s2 2s2 2 p6) and a minimum of two non-bonding electron doublets that help to lose an electron.

• by γ ray irradiation, *R*-*S*-S-R + ionization → R-°S–*S*-R (Favaudon et al., 1990)
• by γ ray or UV activation leading to a hemolytic cleavage,
  o *R*-*S*-S-R + ionization → *R*–S° + *R*–S° (Gillbro, 1974)
  o R° (or ROO°) + R–SH → RH (or ROOH) + *R*–S° (Wongkongkathep et al., 2015; Kobayashi, 2019), with radicals R° or *R*–OO° produced by irradiations.

Thiol radicals, *R*–S°, are very reactive and lead to a high number of *S*-volatiles. Moreover, the chemical changes undergone by the product and the formation of new molecules (such as radiolytic products) are still subject of scientific research and controversy toward consumer's health (Grolichová et al., 2004). In the oxidation mechanism, this step is named “diversification”: after the initiation step that produces radicals (ROO°, HOO° by heat induced radicalization or lipoxygenase catalysis activity; from excited dioxygen, °O–O°, due to photooxidation; by catalysis with Fe or Cu ions; after irradiations), the radicals react with other molecules, then give radical scission reactions that produce a high number of varied volatiles.

**Fig. 3:**
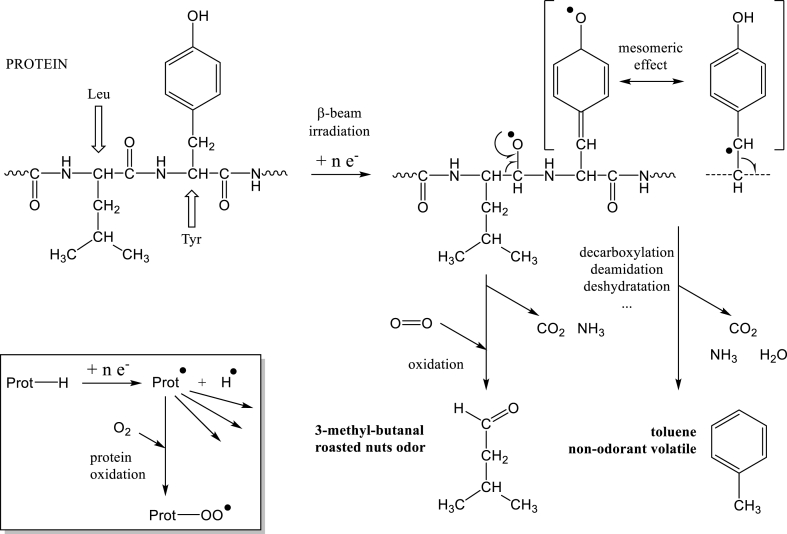
Overview of mechanisms that give marker volatiles from irradiated proteins. Legend: Leu = Leucine, Tyr = Tyrosine.

Oxidation of cysteine produced methanethiol [39] but in Table 1 this component did not increased after irradiation. In proteins, cysteine residues rarely possess a thiol function (R–SH) and are mainly under the form of disulfide function (*R*-*S*-S-R’). These disulfide bonds structure the globular proteins such as plant proteins. As in plant proteins, there are essentially Cys-Cys residues (cystinyl residues) and not Cys residues (cysteynyl residues), this can explain the absence of methanethiol production during oxidation in the present study. Fig. 3 summarizes the oxidation processes of proteins during irradiation of PPI and shows mechanisms of production for two volatile compounds, based on mechanistic hypotheses previously described.

**Fig. 4:**
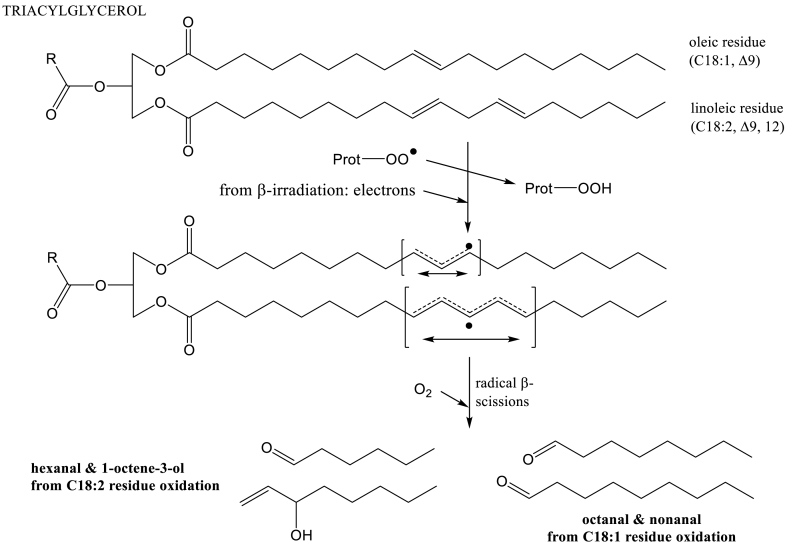
Overview of mechanisms that give marker volatiles from irradiated lipids (triacylglycerols).

The increased amounts of alcohols such as 1-octen-3-ol or 2-ethyl-1-hexanol (Table 1) were probably due to the oxidation of the lipids still present in pea protein isolates, according previous results [34]. Irradiation of PPIs with β-beam led to the appearance of 2-octanone and 3-octanone (except PEA4 for which a great increase was observed). Oxidation of lipids of PPI began during the process of isolation of proteins and continued during drying. Nevertheless, the protein oxidation due to irradiation initiated lipid oxidation [41] that increased the content of volatiles observed after irradiation. Aldehydes and ketones are classical products of the oxidation of triacylglycerols, but the type of the obtained volatile compounds depends on the position of unsaturation of the carbon structure. In crude oil from pea, the content of oleic (C18:1), linoleic (C18/2) and linolenic (C18/3) acid residues are quite equivalent [42]. The oxidation of unsaturated fatty acid residues of triacylglycerols produces marker volatiles specific of ω−9, ω−6, ω−3 fatty acid residues. Octanal, nonanal, decanal, and 2-decenal are volatiles representative of the oxidation of oleic acid residues (C18:1,Δ9; ω−9) of triacylglycerols. Hexanal, 1-octene-3-ol, 2-nonenal, and 2,4-decadienal, 2-octenal come from the oxidation of linoleic acid residues ((C18:2,Δ9, 12; ω−6) and 2-pentenal/2-hexenal from linolenic acid residues (C18:3,Δ9,12,15; ω-3) [43,44]. As reported in Table 1, octanal, 2-octanone, 3-octanone appear, and nonanal, hexanal, 1-octen-3-ol, 2-octenal, 2-hexenal concentration increased after irradiation and these volatile compounds corresponded to the lipids (triacylglycerols) found in PPI. It can be noticed that 2-hexenal concentration did not change after irradiation of PP2 (Table 1), and that 2-nonenal was rarely identified (Table 2). Fig. 4 gives examples of volatiles produced by triacylglycerol oxidation, based on mechanisms described previously [45].

## 4 Conclusions

The data and mechanisms presented in this study showed that electron-beam irradiation of pea protein isolates initiated both protein and lipid oxidations. Generally, lipid oxidation is described as the result of protein oxidation [41,46], but water radical hydrolysis (H_2_O → HO° + H°) due to β-beam irradiation can also initiate directly lipid oxidation.

Ionization had thus a strong impact on the volatile compounds of pea protein isolates, leading to global increase of amounts and to appearance of newly formed compounds. This might result in a complete modification of the odorant profile that could be detrimental for the future use of such products. Indeed, ionization led to the appearance and increase of compounds linked to negative sensory attribute for leguminous protein. Ionization may be not suited to treat leguminous protein as it does not preserve the volatile compound profile of the product and we do not recommend it for the treatment of PPI or other protein concentrate or isolate in the objective to preserve the sensory quality of the product.

As the different chemical families did not evolve the same way, the initial profile in volatile compounds may have had an impact on the evolution caused by ionization.

## Funding

This work was supported by the 10.13039/501100011773Regional Council of Bourgogne – Franche Comté, the 10.13039/501100008530European Regional Development Fund (ERDF), and a grant from Roquette Freres S.A.

## Data availability

The authors do not have permission to share data.

## CRediT authorship contribution statement

**Estelle Fischer:** Writing – original draft, Methodology, Data curation, Conceptualization. **Philippe Cayot:** Writing – original draft, Validation, Formal analysis, Data curation. **Rémy Cachon:** Validation, Supervision, Project administration, Funding acquisition. **Nathalie Cayot:** Writing – review & editing, Writing – original draft, Validation, Supervision, Project administration, Funding acquisition, Conceptualization.

## Declaration of competing interest

The authors declare that they have no known competing financial interests or personal relationships that could have appeared to influence the work reported in this paper.
